# The Dual Role of VEGF in Intracerebral Haemorrhage: From Pathological Mechanisms to Therapeutic Opportunities

**DOI:** 10.31083/RN51435

**Published:** 2026-06-30

**Authors:** Yunlian Niu, Xiaoqian Wang, Yichen Huang, Xuemei Zhang, Yijie You, Peiyuan Ding, Fengbing Sun, Jian Zhang

**Affiliations:** ^1^Department of Neurology, Chongming Hospital Affiliated to Shanghai University of Medicine and Health Sciences, 202150 Shanghai, China; ^2^Department of Neurosurgery, Chongming Hospital Affiliated to Shanghai University of Medicine and Health Sciences, 202150 Shanghai, China; ^3^Ward 4 of the Stroke Center, Chongming Hospital Affiliated to Shanghai University of Medicine and Health Sciences, 202150 Shanghai, China

**Keywords:** cerebral haemorrhage, vascular endothelial growth factor, neuroprotective agents, signal transduction, blood–brain barrier

## Abstract

An intracerebral haemorrhage (ICH) is a general type of haemorrhage in the brain, which arises due to the rupture of cerebral blood vessels. Many molecules have been investigated for their potential ability to modify hemostasis and blood-brain barrier function in ICH. Vascular endothelial growth factor (VEGF) is highly expressed in some diseases and helps induce angiogenesis. VEGF serves as a key mediator of the angiogenic pathway and is now recognized as a major contributor to cerebrovascular diseases; many promising results have been obtained in animal therapeutic experiments. VEGF promotes blood vessel growth (angiogenesis) to increase oxygen and nutrient supply in damaged tissues, and exerts neuroprotective effects in processes including inflammation, apoptosis, neurotrophy, and protein clearance. This review examines the dual roles of VEGF in the pathology of ICH and explores therapeutic targets of VEGF pathways for this disease.

## 1. Introduction

Intracerebral haemorrhage (ICH) is a serious problem around the world, and the main reason is generally high blood pressure [1]. According to the Global Burden of Disease (GBD) Study 2021, stroke was the third leading cause of death in the world, with approximately 7.3 million deaths (accounting for 10.7 per cent of all deaths) and about 160.5 million disability-adjusted life years (DALYs) [2]. Of the 11.9 million incident strokes around the world in 2021, ICH accounted for 28.8% (approximately 3.4 million cases) [2,3]. Although it has a lower incidence, ICH accounts for a disproportionately high share of all stroke-related deaths at 45.6% and almost half of all stroke-related DALYs (49.5%), exceeding the DALY burden of ischaemic stroke (43.8%) [4]. The burden of ICH varies among regions; Oceania (2582.5 per 100,000), Southeast Asia (1976.8 per 100,000) and East Asia have relatively high age-standardized DALYs, whereas high-income areas such as Australasia (126.6), Western Europe (161.2) and high-income North America (221.2) bear a significantly lighter load [5]. About 94 per cent of DALYs lost due to ICH occur in low- and middle-income countries [4]. Hypertension is still the main risk factor and accounts for more than 50% of the ICH burden across all socio-demographic groups [4,5].

Currently, there are only a few treatment options for ICH. The 2022 American Heart Association/American Stroke Association (AHA/ASA) guidelines advocate for early blood pressure management (aiming for systolic blood pressure <140 mmHg within 2 hours of onset), discontinuation of anticoagulation for eligible individuals,and surgical evacuation of intracranial haematoma in cases with large or expanding haematomas [6]. Surgical interventions, whether open craniotomies or minimally invasive methods, have not yielded the same improvements in function across all large randomised controlled trials. The Surgical Trial in Intracerebral Haemorrhage(STICH) trials did not find an advantage for early surgery over conservative management in supratentorial ICH [7]. Although the Minimally Invasive Surgery plus Alteplase for Intracerebral Haemorrhage Evacuation (MISTIE) III trial showed that minimally invasive surgery plus thrombolysis was safe, it did not meet the first endpoint for functional improvement [8]. The first kind of treatment is to control high blood pressure through support and observation of the brain for any changes or other diseases. Importantly, no pharmaceutical product has been approved to date for the purpose of neuroprotection or acceleration of haematoma reduction in ICH [9]. The therapeutic gap suggests that new targets for therapy need to be identified, and vascular endothelial growth factor (VEGF) has shown dual roles in angiogenesis and neuroprotection, as well as glymphatic-mediated haematoma resolution; therefore, it is a promising candidate in this process.

Given the high mortality and significant morbidity and long-term disability caused by it, ICH has gradually drawn the attention of scholars around the world. ICH should not be viewed as an easy-to-reverse phenomenon; rather, it triggers an extended pathological process through multiple pathways [10]. They include the formation of a haematoma and local cerebral oedema; as well as cellular ischaemia, hypoxia and metabolic disorders in the brain tissue; all of which promote nerve injury.

To reduce damage to neural function caused by ICH, many scholars have put forward various interventions that aim to improve cellular ischaemia and hypoxia, as well as alterations in brain tissue metabolism [11]. Given the above reasons, angiogenesis has gradually been used to improve blood supply in the damaged region and thus supply more oxygen and nutrients. Decrease damage to the neurons significantly. Angiogenesis has, in fact, been shown in various studies to promote agood prognosis for cerebral haemorrhage.

VEGF is required for angiogenesis, the proliferation of endothelial cells and the formation of new blood vessels [12,13,14]. However, high expression of VEGF has also been associated with the pathology of hypertensive ICH, and it may be causing increased inflammation, vascular permeability and perihematomal oedema, as shown by Yang and Shao [14]. Both are protective and potentially damaging; thus, VEGF is a particularly interesting but also problematic therapeutic target in ICH.

Review of the two functions of VEGF in ICH is presented here: protective effects, including the promotion of angiogenesis, neuroprotection and glymphatic clearance of haematomas; damaging effects caused by compromise of the blood-brain barrier (BBB) and vasogenic oedema; and regulatory mechanisms governing these context-dependent activities.

## 2. Literature Search Methods

This narrative review will present the latest data on the regulatory system and treatment function of VEGF in ICH. Based on the data provided by PubMed (https://pubmed.ncbi.nlm.nih.gov/) and Web of Science (https://www.webofscience.com/), We have collected some relevant articles that have been released since January 2000. The following search terms were used in combination: “vascular endothelial growth factor” OR “VEGF”, “intracerebral haemorrhage” OR “ICH” OR “cerebral haemorrhage”, “angiogenesis”, “neuroprotection”, “blood–brain barrier”, “glymphatic system”, and “VEGF receptor”. Some related articles were found by manually examining the reference lists of the retrieved publications.

The following articles were selected for inclusion: (1) they were published in English; (2) they focused on VEGF biology, signaling pathways or therapeutic targeting in the context of ICH or related cerebrovascular diseases; and (3) they were original research articles, clinical studies or high-quality reviews. Studies were excluded if they were only published as conference abstracts, case reports or other non-peer-reviewed works. Given that this is a review of narratives, no standardised quality evaluation instruments or Preferred Reporting Items for Systematic Reviews and Meta-Analyses (PRISMA) flowcharts were used. Evidence sources — such as *in vitro* experiments, *in vivo* animal models, *ex vivo* human tissue studies and clinical investigations — are all explicitly listed in the paper to show the strength of evidence supporting each core result.

## 3. Regulatory Role of VEGF in the Pathological Process of ICH

VEGF promotes the formation of endothelial cells. It increases vascular permeability, damages the extracellular matrix, migrates and proliferates endothelial cells, and thus promotes angiogenesis [15].

**Fig. 1.**
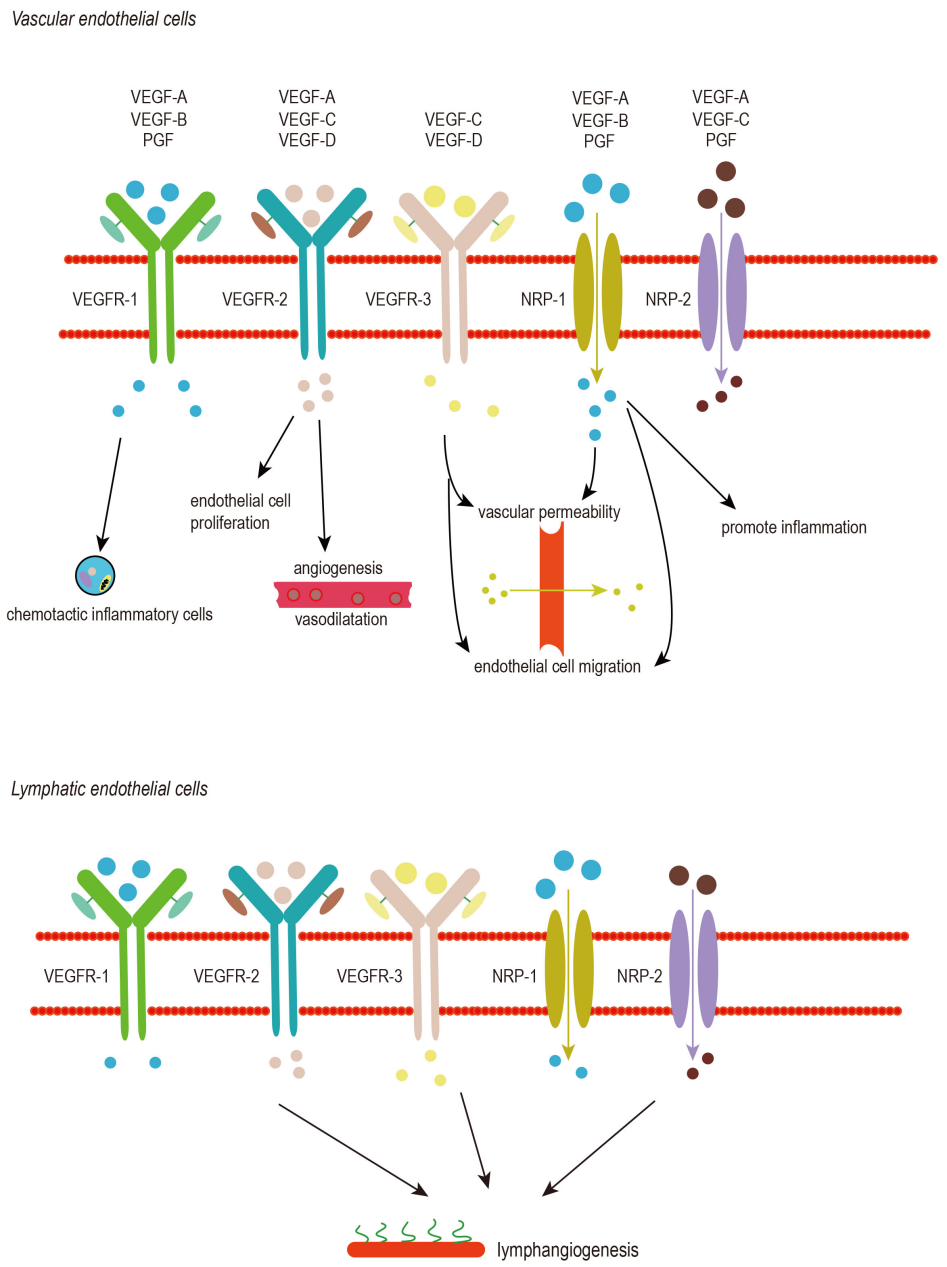
**The distribution and function of VEGF and its receptors**. Shown here is a schematic overview of VEGF family members—VEGF-A, -B, -C, -D, -E, -F, and placental growth factor (PGF)—and how they pair with their receptors. VEGF-A recognizes both VEGFR-1 and VEGFR-2, whereas VEGF-B and PlGF bind only VEGFR-1. VEGF-C and VEGF-D are a bit different: they interact with VEGFR-2 as well as VEGFR-3. VEGFR-1 and VEGFR-2 are found mainly on blood endothelial cells, where they regulate angiogenesis andvascular permeability. VEGFR-3, in contrast, is largely restricted to lymphatic endothelial cells and drives lymphangiogenesis. What sets VEGF-C and VEGF-D apart from the rest is their dual-receptor activity—on vascular endothelium they engage VEGFR-2 to promote angiogenesis, and on lymphatic endothelium they use VEGFR-3 to mediate lymphangiogenesis, making them shared ligands across both vessel systems. VEGF, vascular endothelial growth factor; NRP-1, neuropilin-1; NRP-2, neuropilin-2.

The members of the VEGF family include VEGF-A, VEGF-B, VEGF-C, VEGF-D, VEGF-E, VEGF-F, and placental growth factor (PGF) [16,17]. VEGF can generate several types through exon splicing, including VEGF-121, VEGF-165, VEGF-189 and VEGF-206. Among them, VEGF-165 has the highest physiological activity and is also the most common [18]. VEGFRs are mainly VEGFR-1, VEGFR-2 and VEGFR-3, all of which are receptor tyrosine kinases. VEGF binds to VEGFR-1 and VEGFR-2, and is thus involved in chemotaxis and inflammation. It binds to VEGFR-3 and thus induces lymphangiogenesis. VEGFR-2 is the main signaling receptor, has higher tyrosine kinase activity than VEGFR-1, and conducts intracellular signal transmission for angiogenesis and neuroprotection [19,20,21]. The distributions and functions of the different types of VEGF vary. For more details, see Fig. 1.

## 4. VEGF-Related Pathways in Intracerebral Haemorrhage: An Integrated Signaling Network

The cascade of downstream pathways for VEGF signalling in ICH is a network rather than an isolated linear path. Upon binding to its receptors, VEGF organises a series of changes in cells that can be classified into two main branches emanating from a central VEGFR-2-phospholipase C gamma (PLCγ) node: (1) the the phosphoinositide 3-kinase (PI3K)/protein kinase B (Akt)-endothelial nitric oxide synthase (eNOS) pathway, which mainly promotes cell survival, vasodilation and the formation of blood–brain barrier; and (2) the PLCγ-Ca^2+^-cyclooxygenase (COX)-2 route, which leads to actin cytoskeleton rearrangement, endothelial cell migration and an angiogenic amplification loop via prostanoids. These modules are not independent but work together mechanically, and their relative weights change according to the stage of ICH.

### 4.1 A Central Branch Point: VEGFR-2–PLCγ Divergence Into Ca^2+^ and DAG–PKC Signaling

VEGF-A binds to VEGFR-2 to form a dimer, which then phosphorylates its own sites for the recruitment of other signalling proteins. Among them, PLCγ is recruited and activated to hydrolyze phosphatidylinositol 4,5-bisphosphate (PIP_2_) into the two second messengers inositol 1,4,5-trisphosphate (IP_3_) and diacylglycerol (DAG). This reaction is the splitting point of the two linked signalling pathways. IP_3_ triggers the release of Ca^2+^ from endoplasmic reticulum stores, and the resulting rise in cytosolic Ca^2+^ serves two purposes: it directly activates eNOSto boost the generation of nitric oxide (NO) and strengthens the PI3K/Akt–eNOS pathway mentioned above in references [22,23,24]; and it induces changes in the actin–myosin cytoskeleton required for endothelial cell migration during angiogenic sprouting. DAG activates protein kinase C (PKC) through elevated cytosolic Ca^2+^, and in parallel, DAG synergistically with elevated cytosolic Ca^2+^ activates the mitogen-activated protein kinase (MAPK)/ extracellular signal-regulated kinase(ERK) cascade to drive transcriptional upregulation of COX-2 via activator protein-1 (AP-1) and nuclear factor kappa-light-chain-enhancer of activated B cells (NF-κB) [25,26]. Thus, PLCγ-mediated generation of IP_3_ and DAG simultaneously triggers Ca^2+^-dependent migration and PKC/MAPK-induced COX-2 expression to establish a coordinated angiogenic response.

### 4.2 The PI3K/Akt–eNOS Axis: Survival Signaling, Vasodilation and Glymphatic Support

At the same time as the activation of PLCγ, VEGF-A/VEGFR-2 signaling recruits PI3K to the receptor complex. PI3K generates phosphatidylinositol (3,4,5)-trisphosphate (PIP_3_) at the plasma membrane and recruits Akt through its pleckstrin homology domain to activate Akt by phosphorylation [27,28]. Activated Akt phosphorylates several substrates that have different functions in ICH. First, phosphorylation of eNOS at Ser1177 increases NO production to promote vasodilation and improved local perfusion, and this effect is further enhanced by Ca^2+^-mediated activation of eNOS initiated through the PLCγ branch described above [23,27,28]. Secondly, by phosphorylating and thus inhibiting pro-apoptotic proteins such as Bad and caspase-9, neuroprotection has been achieved through the suppression of neuronal apoptosis [25]. Third, mechanistic target of rapamycin (mTOR) is activated to promote the survival and protein synthesis of endothelial cells in angiogenesis.

The PI3K/Akt pathway is another one of the first targets of VEGFR-3 for the signal from VEGF-C. As shown by Liao and others [12], simvastatin treatment after experimental ICH activates the VEGF-C/VEGFR-3/PI3K/Akt pathway, protects the structure and function of the glymphatic system by preserving aquaporin-4 polarization on astrocytic end-feet, restricts the expansion of perivascular spaces, and promotes the proliferation of meningeal lymphatic vessels. This VEGFR-3–PI3K/Akt–eNOS signaling module has a certain function, and unlike VEGFR-2, it is less coupled with pro-permeability Src pathways; therefore, it avoids the BBB-disrupting effects that occur after acute activation of VEGFR-2 (see Section 6 for a detailed analysis of the temporal dynamics).

### 4.3 The COX-2 Pathway: Prostanoid-Mediated Amplification of Angiogenesis

The PKC/MAPK/ERK pathway is activated by DAG at the VEGFR-2–PLCγ bifurcation point and then leads to increased transcription of COX-2. The following increase in COX-2 enzyme activity promotes the production of several important prostaglandins, such as prostaglandin E_2_ (PGE_2_) and prostacyclin (PGI_2_) [26,29,30]. Lipid mediators are autoregulatory and paracrine, bind to the corresponding G protein-coupled receptors (EP and IP receptors), and thus induce vasodilation, increase vascular permeability and modify inflammation. COX-2-derived prostanoids are in two different states in the context of ICH, which correspond to the time lag of VEGF signalling: excessive COX-2 activation in the acute stage may lead to pathological vascular permeability and inflammation, while COX-2-derived PGE_2_ and PGI_2_ are needed for organising angiogenic remodelling and recruiting endothelial progenitor cells to the perihematoma in the recovery stage [26,31].

### 4.4 Pathway Integration and Phase-Dependent Functional Shifts

The three signaling modules mentioned above are mechanistically linked at multiple levels: PI3K/Akt–eNOS, Ca^2+^-dependent migration, and COX-2-mediated prostanoid synthesis. The IP_3_–Ca^2+^ arm of the PLCγ branch enhances eNOS activity stimulated by PI3K/Akt, and the DAG–PKC arm induces COX-2 expression to maintain angiogenesis. Conversely, NO produced by the PI3K/Akt–eNOS axis can also regulate COX-2 activity and thereby create a bidirectional communication loop between the survival and prostanoid modules.

**Fig. 2.**
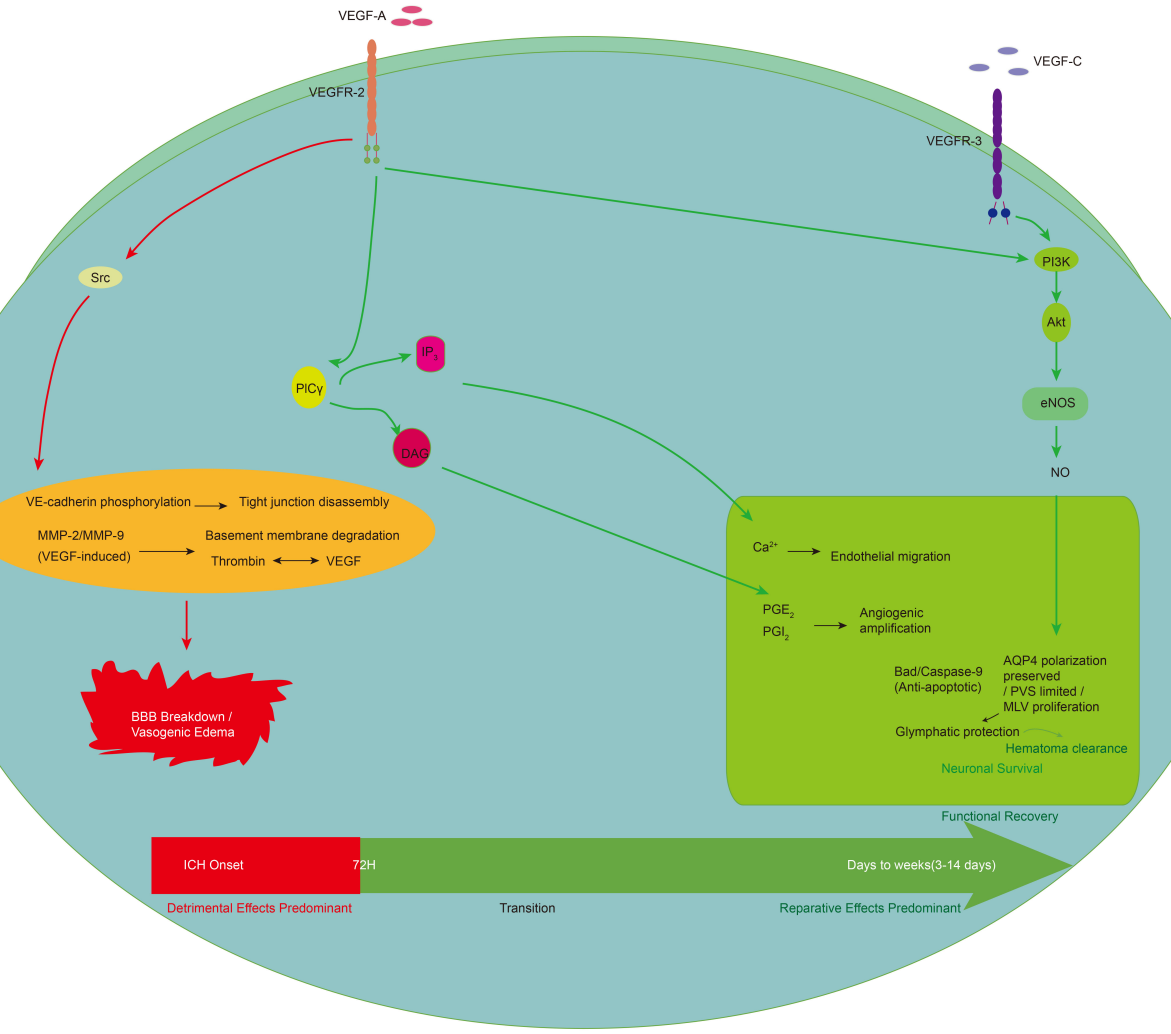
**Integrated VEGF signaling network in intracerebral haemorrhage**. When VEGF-A binds VEGFR-2, it activates PLCγ, which then generates IP_3_ and DAG. The IP_3_-triggered Ca^2+^ release helps endothelial cells migrate and, through crosstalk (dashed lines), gives a boost to PI3K/Akt–eNOS signaling. On a parallel track, DAG switches on the PKC/MAPK/ERK cascade, leading to COX-2 induction and the production of prostanoids such as PGE_2_ and PGI_2_. Early on, in the acute phase (left red box), VEGFR-2/Src signaling phosphorylates VE-cadherin and, together with MMP-mediated degradation of tight junctions, opens up the blood–brain barrier and causes vasogenic oedema; a thrombin–VEGF positive-feedback loop compounds this disruption. Later, during the subacute–chronic phase (right green box), the picture changes—PI3K/Akt–eNOS and PLCγ–Ca^2+^ pathways now support angiogenesis. At the same time, VEGF-C/VEGFR-3/PI3K/Akt signaling helps preserve glymphatic function by maintaining AQP4 polarity, perivascular space (PVS) integrity, and meningeal lymphatic proliferation, which speeds up haematoma clearance and protects neurons. The time arrow at the bottom emphasizes this functional switch from injury to repair as intracerebral haemorrhage evolves. PLCγ, phospholipase Cγ; IP_3_, inositol 1,4,5-trisphosphate; DAG, diacylglycerol; PI3K, phosphatidylinositol 3-kinase; Akt, protein kinase B; eNOS, endothelial nitric oxide synthase; PKC, protein kinase C; MAPK, mitogen-activated protein kinase; ERK, extracellular signal-regulated kinase; COX-2, cyclooxygenase-2; PGE_2_, prostaglandin E_2_; PGI_2_, prostacyclin; VE, vascular endothelium; MMP, matrix metalloproteinase; AQP4, aquaporin-4.

The function of the combined network will change based on the period of ICH (as shown in Section 7). In the acute stage, an increase in VEGF-A primarily activates the VEGFR-2–Src pathway, inhibits the PI3K/Akt survival module, and promotes VE-cadherin phosphorylation; thus, tight junctions are disassembled and basement membrane degradation by matrix metalloproteinases (MMPs) occurs, leading to BBB disruption and vasogenic oedema [22,24,32,33]. As ICH moves into the subacute and chronic stages, the acute inflammatory response subsides and the haematoma debris is cleared; at this time, the PI3K/Akt-eNOS and Ca^2+^-COX-2 angiogenic modules become functionally dominant to drive microvascular neovascularisation, glymphatic restoration, and neuronal protection. The VEGF-C/VEGFR-3 axis acts mainly through PI3K/Akt and is only weakly connected to the pro-permeability Src pathway in promoting repair. As shown in Fig. 2, the overall effect of VEGF signalling in ICH is not caused by the activation of a single pathway but rather by the phased cooperation of all of them.

## 5. VEGF and Neuroprotection

In recent years, the neuroscience community has been studying the non-vascular functions of VEGF more intensely. According to the findings of the ICH, VEGF has shown many other roles in the past that promote angiogenesis, but also as a potent neuroprotective and neurotrophic factor for neurons. Experimentally determined, the VEGF family can also protect both neurons and all regions within the neurovascular unit, such as VEGF-C.

VEGF is needed to run the glymphatic system and can help the brain recover from an ICH. As shown by Liao et al. [12], simvastatin treatment after experimental ICH activates the VEGF-C/VEGFR-3/PI3K/Akt signalling pathway. Actively maintain the structure and function of the glymphatic system by keeping aquaporin-4 polarised at the astrocytic end-feet, preventing the expansion of the perivascular space, and promoting the proliferation of meningeal lymphatic vessels. At the bottom of this pathway, an increase in the expression of endothelial eNOS will produce NO; thus, it will diffuse into the cerebrospinal fluid and be involved in the glymphatic system. VEGF-C may enhance the function of the glymphatic system to reduce bleeding in the brain, lower iron accumulation, and thus reduce damage to neurons at a functional level.

The above results have expanded our knowledge of the neuroprotective function of VEGF in ICH to include non-neuronal cells. VEGF-C will be released to induce anti-apoptotic and pro-neurotrophic effects and also regulate the clearance mechanism. Protection of the Perivascular Drainage Path can reduce the amount of toxic blood causing secondary brain damage. Therefore, a new way of treating ICH has been introduced by combining vascular signals and the glymphatic system to induce VEGF-C/VEGFR-3. Therefore, future translational efforts should focus on ICH-relevant delivery strategies to specifically activate VEGFR-3 signaling in the glymphatic system and enhance neuroprotection under reduced off-target angiogenic risks.

## 6. VEGF and the BBB

In line with the ICH guidelines, there is damage to the endothelial cells of blood vessels, and consequently, serum, plasma proteins and other components in the blood leak into the perivascular space to form extracellular fluid (vasogenic oedema of the brain). This oedema can worsen local microcirculation diseases, further increase the ischemic and hypoxic state, and lead to progressive neuronal damage [34]. At the same time, the exposed basement membrane of damaged endothelial cells can promote platelet adhesion and migration [35,36]. At the same time, platelets specifically bind to von Willebrand factor (vWF), fibronectin and collagen via their surface receptors and are activated; activated platelets then release a large amount of VEGF, which not only increases BBB permeability further but also forms a positive feedback loop to recruit more platelets for aggregation [37]. Platelet aggregates can rapidly block small blood vessels, cause local microcirculatory disorders and ischaemia, and also increase the expression of VEGF to promote vasogenic oedema [38,39]. Endothelial injury can also start the coagulation cascade, and the final product of this pathway, thrombin, is capable of substantially increasing vascular permeability by causing remodelling and contraction of the cytoskeleton in endothelial cells. Research has shown that thrombin is a strong inducer of VEGF release, and VEGF can also promote the conversion of prothrombin to thrombin through a non-classical pathway and enhance the catalytic activity of thrombin by as much as 5 times [38]. Therefore, thrombin and VEGF have formed a dual positive-feedback regulatory mechanism to drive the persistent increase in BBB permeability after ICH.

## 7. VEGF’s Dual Roles in ICH: Temporal Changes and Dose-Dependent Effects of Context-Dependent Regulation

VEGF is generally known to be protective of the central nervous system, but not all VEGF has shown this benefit in the context of ICH. Based on the above evidence, it can be concluded that VEGF has dual functions in different environments; namely, it is detrimental in a disease state and advantageous during the latter stages of development in damaged tissues. The two different times, combined with different functions at different doses, are major causes of varying responses to VEGF therapy.

### 7.1 Acute Phase (0–72 hours): Harmful Effects Driven by Vascular Permeability

VEGF levels rise quickly during the hyperacute and acute periods of ICH, as early as 24 hours after haemorrhage, and this increase is mainly associated with harmful effects [40]. VEGF’s main way to cause harm in the short run is by breaking down the BBB. VEGF binds to VEGFR-2 on cerebral endothelial cells and triggers a downstream Src kinase-mediated phosphorylation of VE-cadherin, leading to the disassembly of adherens junctions and internalization of tight junction proteins, including claudin-5 and occludin [22,41]. At the same time, VEGF also increases the expression of matrix metalloproteinases MMP-2 and MMP-9, thus damaging the endothelial basement membrane and further impairing vascular integrity [32,42].

VEGF-induced BBB disruption promotes vasogenic oedema and is thus one of the primary causes of secondary brain injury and poor prognosis in ICH. VEGF also participates in a bidirectional positive feedback loop with thrombin: thrombin, produced by the coagulation cascade triggered after damage to the endothelium, stimulates the release of VEGF, and simultaneously, VEGF promotes the conversion of prothrombin to thrombin and enhances the catalytic activity of thrombin by about 5-fold [38]. The thrombin-VEGF amplification loop keeps expanding the BBB leak and thus increases surrounding oedema and microcirculatory failure. Another type of acute-phase damage is the increase in platelet activation and aggregation; therefore, there is a risk of microvascular obstruction and tissue ischaemia [43,44]. Therefore, in the first 72 hours after ICH, the pathologically elevated VEGF mainly functions as a pro-edematous and pro-coagulopathic factor to drive secondary injury cascades. Most of the mechanistic data come from *in vitro* experiments and *in vivo* studies in rodents for ICH [22,32,38], and Babkina and others have shown that early VEGF elevation can also be observed in patients [40].

### 7.2 Subacute to Chronic Phase (3-14 Days and Later): Transition to Repair Functions

As ICH moves into the subacute and chronic phases, VEGF will have lost some of its beneficial effects for coordination of the body’s response to injury and repair and will be more detrimental. At this time, changes in the local molecular microenvironment have occurred, such as reduced acute inflammation, removal of haematoma components, and alterations in the expression of VEGF receptors and downstream signaling effectors.

A typical repair mechanism that has begun to emerge at this time is VEGF-induced activation of the glymphatic system. As shown by Liao et al. [12], activation of the VEGF-C/VEGFR-3/PI3K/Akt signaling pathway protects the structure and function of the glymphatic system by maintaining the polarisation of aquaporin-4 (AQP4) on astrocytic end-feet, restricting perivascular space expansion, and promoting proliferation of meningeal lymphatic vessels. Downstream, an increase in the expression of eNOS will maintain the homeostatic regulation of nitric oxide in the local area, thus facilitating the entry and stability of glymphatic transport for cerebrospinal fluid. VEGF-C drives glymphatic preservation to accelerate the clearance of haematomas and reduce iron deposition, thus inhibiting neuronal apoptosis via a clearance-dependent neuroprotective pathway separate from that promoted by VEGF.

At the same time, VEGF promotes angiogenesis in the perihematomal area by activating VEGFR-2 and subsequently the PI3K/Akt–eNOS and PLCγ–Ca^2+^ pathways to stimulate endothelial cell proliferation, migration and tube formation. Microvascular neovascularisation is formed in this way to restore blood supply to the ischaemic penumbra, enhance oxygen and nutrient delivery, and reduce secondary infarction [17,45]. Moreover, the Janus kinase 2/signal transducer and activator of transcription 3 (JAK2/STAT3) signaling pathway is activated after ICH and induces prolonged overexpression of VEGF in the perihematomal area; thus, it integrates early inflammatory signals to promote both later angiogenesis and neurogenesis for structural and functional repair [46]. Collectively, these multiple types of repair will help improve the neurological state in the face of modified VEGF signaling.

### 7.3 Dose-Dependent Divergence of VEGF Effects

VEGF signaling also has a function that depends on how concentrated it is locally in space and time. Low- to moderate-level VEGF preferentially activates the PI3K/Akt survival pathway, promoting the survival of endothelial cells and angiogenesis with maintained barrier integrity, as well as protecting neurons. On the other hand, a high level of VEGF in the acute stage of severe ICH can lead to unchecked activation of Src kinases and abnormal phosphorylation of VE-cadherin, resulting in a loss of vascular integrity that exceeds the advantages of newborn angiogenesis [25]. As shown in the above figure, a large dose will not help recovery; therefore, if a high level of exogenous VEGF is applied for an extended period of time, it may damage the injured area instead of promoting healing. Therefore, the next therapeutic strategies will need to regulate VEGF signalling more accurately and maintain a high enough concentration to activate the repair pathway without reaching the threshold for pathological vascular leakage.

### 7.4 Summary of the Dual Roles of VEGF in ICH

Together, the role of VEGF in ICH is not best understood as a binary “beneficial or harmful” characteristic, but rather as a time- and dose-dependent continuum. As shown in Table 1 (Ref. [12,17,22,25,32,33,38,39,40,42,45,46,47]), VEGF has two functions at different times in the acute and recovery periods, and the specific effects, supporting molecular mechanisms and functional results, are detailed below. Based on the above analysis, personalised differentiation strategies for VEGF inhibitors in ICH therapy are to be developed according to different stages of disease progression and should include spatiotemporal control techniques.

**Table 1.** **Dual role of VEGF in ICH**.

Phase	Role	VEGF species/Receptor	Mechanism & Signaling pathway	Functional outcome	References
Hyperacute (0–24 h)	Minimal involvement	VEGF-A/VEGFR-2	No significant change in serum VEGF-A or VEGFR-2 levels	VEGF signaling not yet activated; diagnostic window	Babkina et al., 2022 [40]
Acute (24–72 h)	Detrimental	VEGF-A/VEGFR-2	Src/VE-cadherin phosphorylation → tight junction disassembly (claudin-5, occludin)	BBB breakdown, vasogenic oedema	Argaw et al., 2009 [22]; Wang et al., 2021 [46]
			MMP-2/MMP-9 upregulation → basement membrane degradation	Increased vascular permeability, oedema exacerbation	Shen et al., 2018 [32]; Ismael et al., 2020 [42]; Guan et al., 2025 [33]
			Thrombin–VEGF positive feedback loop	Sustained BBB hyperpermeability, coagulopathy	Jiang et al., 2022 [38]; Gupta et al., 2024 [39]
		VEGF-A	Platelet activation and aggregation	Microvascular occlusion, local ischaemia	Gupta et al., 2024 [39]
Subacute-Chronic (3–14 d)	Beneficial	VEGF-C/VEGFR-3	PI3K/Akt → eNOS → NO → glymphatic protection (AQP4 polarization, PVS preservation, MLV proliferation)	Haematoma clearance, reduced iron deposition, attenuated neuronal apoptosis	Liao et al., 2024 [12]
		VEGF-A/VEGFR-2	PI3K/Akt–eNOS and PLCγ–Ca^2+^ pathways → endothelial proliferation, migration, tube formation	Angiogenesis, improved perihematomal perfusion	Wang et al., 2020 [17]; Zeng et al., 2024 [45]
			JAK2/STAT3 → sustained VEGF expression	Microvascular remodeling, coupling of inflammation to repair	Wang et al., 2021 [46]
			PI3K/Akt → Bcl-2 upregulation, caspase inhibition; neurogenesis (SVZ, DG)	Neuronal survival, synaptic plasticity, axonal sprouting	Sun et al., 2003 [47]
Dose-Dependent	Beneficial (low–moderate dose)	VEGF-A/VEGFR-2	PI3K/Akt survival signaling	Controlled angiogenesis with preserved barrier integrity	Geiseler & Morland, 2018 [25]
	Detrimental (high dose)		Excessive Src activation → pathological VE-cadherin phosphorylation	Disorganized vascular permeability, oedema	Geiseler & Morland, 2018 [25]

Abbreviations: AQP4, aquaporin-4; BBB, blood–brain barrier; eNOS, endothelial nitric oxide synthase; JAK2/STAT3, Janus kinase 2/signal transducer and activator of transcription 3 signaling pathway; MLV, meningeal lymphatic vessel; MMP, matrix metalloproteinase; NO, nitric oxide; PVS, perivascular space; SVZ, subventricular zone; DG, dentate gyrus.

## 8. Therapeutic Targets for VEGF in Intracerebral Haemorrhage: Strategies, Clinical Progress and Challenges

VEGF has the function of damage and healing in the subacute stage after ICH, so the control strategy for this signalling pathway should focus on space and time rather than broadly shutting down or broadly enabling it. The following are the current trends in VEGF-targeting strategies, relevant clinical data, and main problems of translating preclinical results to the clinic.

### 8.1 Therapeutic Measures

#### 8.1.1 Indirect Modulation via Statins

Simvastatin and Atorvastatin are now the most advanced clinical treatments for ICH that can increase protective VEGF signaling indirectly. Preclinical studies have shown that simvastatin activates the VEGF-C/VEGFR-3/PI3K/Akt pathway to promote the preservation of glymphatic function and rapid reduction of haematomas [12]*. *Atorvastatin has shown good results in reducing haematoma in people with long-standing subdural haemorrhages in several randomised studies, and it is generally well tolerated [48,49]. Atorvastatin can also be combined with craniocervical manual lymphatic drainage to enhance the function of glymphatic clearance [50]. Based on the above results, statins have already been approved for clinical use and are known to be safe; thus, they offer a simple, translatable strategy for modulating the VEGF pathway in ICH. However, the ideal time to start, amount, and selection criteria for patients receiving statin therapy after acute ICH have not been determined by prospective studies.

#### 8.1.2 VEGFR-3-Selective Activation

VEGF-C/VEGFR-3 constitutes a favorable therapeutic target: VEGF-C acts as the ligand for receptor VEGFR-3, and their interaction only activates the PI3K/Akt pathway instead of the pro-permeability Src pathway [12]. Receptor-level selectivity of this type can achieve glymphatic protection and haematoma clearance without the BBB-disrupting effect of VEGFR-2 activation. Intracisternal injection of VEGF-C has been shown in preclinical studies to stimulate meningeal lymphatic growth and improve the elimination of a haematoma in ICH models [51]. The development of VEGFR-3-selective agonists or VEGF-C-based biologics may offer a safer option compared with the broad activation of VEGFR-2.

#### 8.1.3 Designed Delivery Platforms

High-end Delivery systems are being developed to achieve spatiotemporal control of VEGF signalling. These include adeno-associated virus (AAV) vectors with inducible promoters for conditional VEGF expression, BBB-penetrating nanoliposomes for targeted central nervous system (CNS) delivery, and stimulus-responsive fusion proteins that release active VEGF only in specific microenvironments [12,52]. These platforms aim to limit the activity of VEGF to the recovery stage and the perihematomal region to reduce off-target angiogenic risks and acute pro-edematous effects.

#### 8.1.4 Combinatorial Strategies

Given the contrasting roles of VEGF in the different phases of ICH, a biphasic treatment approach is likely more suitable: acutely focus on reducing oedema and stabilizing the BBB (possibly employing short-term anti-VEGF treatments to limit permeability), and subsequently, at a later time, slowly trigger VEGF-C/VEGFR-3 or VEGFR-2-mediated regenerative signals to promote angiogenesis, glymphatic clearance, and neurovascular remodelling.

### 8.2 Clinical Progress

The clinical evidence mentioned above is the result of randomised controlled trials, systematic reviews and meta-analyses, and observational cohort studies [48,49,53].

No VEGF-targeted therapy has been approved for ICH so far. There have also been some changes in the clinic. Statins have been used in ICH research, and based on observational studies, before an ICH episode, some people were on statins and had better outcomes and fewer deaths [53]. The randomised clinical trial of atorvastatin for chronic subdural haematoma showed a significant reduction in haematoma volume and a decreased need for surgery, thus providing proof-of-concept for statin-mediated enhancement of haematoma clearance [48,49]. Bevacizumab and other anti-VEGF drugs are often used in cancer and eye diseases to inhibit the VEGF pathway; therefore, it has been shown that the VEGF pathway is clinically accessible. Given the presence of cerebrovascular disease, research has been conducted on anti-VEGF drugs to reduce the permeability of the BBB and cerebral oedema; early inhibition of VEGF has been shown to mitigate BBB damage in stroke models [54]. However, given the risk of obstructing the following repair angiogenesis, the translation of such approaches to ICH needs to be done cautiously in terms of timing.

### 8.3 Therapeutic Problems

Several major problems need to be solved before VEGF-targeted drugs can be used clinically for small-vessel haemorrhage.

#### 8.3.1 Therapeutic Window

The first problem is how to determine the best time to treat. VEGF inhibition is too early and will block the good-repair signal; otherwise, excessive VEGF activity in the acute stage will cause oedema and coagulopathy. It is necessary to find reliable biomarkers for the shift from the acute injury stage to the recovery stage and thus personalise VEGF-targeted therapy.

#### 8.3.2 Delivery and Biodistribution

Delivery to the target area in the perihematoma and a reduction in systemic exposure of VEGF-modulating agents still need to be optimised. Although partially impaired by ICH, the BBB is still relatively complicated and unstable, thus inhibiting the delivery of drugs. Invasive ways to treat may work well in animal studies, but they are not yet suitable for people.

#### 8.3.3 Dose Adjustment

As shown in Section 7.3, the functional results of VEGF signalling vary with different doses. To reduce the release of VEGF at the local site and avoid damage to other cells in a non-uniform way, small increments of the treatment may be added over a longer period.

#### 8.3.4 Patient Heterogeneity

ICH patients vary significantly in the amount of haematoma, location, age, co-morbidities and genes. The above conditions will reduce the endogenous VEGF response and, thus, lessen the healing effect of VEGF-modulating treatment. Based on the biomarkers of stratification, such as the levels of serum VEGF-A and VEGFR-2 measured by Babkina et al. [40], a subgroup of patients with good responses to specific VEGF-targeted therapies can be identified.

#### 8.3.5 Safety Problems

Long-term activation of VEGF carries the risk of promoting tumour angiogenesis, worsening atherosclerotic plaque neovascularisation, and inducing pathological vascular proliferation. An extended period of safety research will be conducted on non-human models and a selection process for the clinical trial subjects will be carefully implemented.

### 8.4 Links Between Current ICH Therapies and VEGF Modulation

Besides the development of VEGF-targeted strategies, some existing clinical interventions for ICH may also be related to VEGF signalling pathways.

Antihypertensive Agents. Angiotensin-converting enzyme (ACE) inhibitors and angiotensin II receptor blockers (ARBs) are now first-line treatments for acute hypertension in ICH, and they have been shown to reduce perihematomal oedema and mortality in patients with hypertensive ICH [55]. Preclinical studies in ICH rat models have shown that ARB treatment can reduce brain oedema, inflammatory mediators and oxidative stress in the perihematomal region [56]. Angiotensin II is a well-known inducer of VEGF expression via the AT1 receptor-mediated signalling pathway and, at the same time, alters BBB permeability by activating the AT1 receptor on brain endothelial cells [57]. Therefore, it is proposed that some of the clinical benefits of ACE inhibitors and ARBs in ICH may be due to the indirect reduction of VEGF-induced vascular permeability and oedema.

#### 8.4.1 Hemostatic Therapy

Tranexamic acid is an antifibrinolytic agent that has been studied in the TICH-2 randomised controlled trial for ICH; it can reduce the size of a haematoma and early mortality but has not improved 90-day functional outcomes significantly [58]. The STOP-MSU phase 2 trial also reported that intravenous tranexamic acid within 2 hours of the onset of ICH symptoms did not significantly reduce haematoma growth [59]. Plasmin is the target of tranexamic acid, and it is known that plasmin activates matrix metalloproteinases such as MMP-2 and MMP-9, which are downstream effectors of VEGF-mediated BBB disruption [38]. Tranexamic acid inhibits plasmin and stabilizes the fibrin clot to reduce the effect of VEGF-induced vascular permeability near the bleeding site; however, due to the inconsistent results of completed trials, its clinical applicability in this way has not yet been determined.

#### 8.4.2 Surgical Evacuation

Haematoma removal via craniotomy or other minimally invasive means will alter the local microenvironment, thus reducing the expansive effect of VEGF promotion by perimeningeal oedema. MISTIE III trial shows that minimally invasive surgery and thrombolysis reduce the extent of perihematomal oedema [8]. Attenuation of perihematoma oedema after surgical evacuation may be partially due to reduced VEGF-mediated disruption of the BBB and inflammatory signalling, but a direct mechanism by which haematoma evacuation modulates the VEGF pathway has not been identified in clinical studies.

#### 8.4.3 Statins

As shown in Section 8.1, statins are currently advanced clinical therapeutic agents for treating ICH. Preclinical studies have shown that simvastatin directly activates the VEGF-C/VEGFR-3/PI3K/Akt lymphangiogenic pathway to preserve glymphatic function and accelerate haematoma clearance in experimental ICH models [12]. Clinical trials have also demonstrated that atorvastatin can help reduce the extent of a haematoma in chronic subdural haematoma [48,49], as shown in Section 8.2.

## 9. Conclusion

ICH is still a serious stroke type for which no drug treatment has been developed, so new treatment targets urgently need to be found. VEGF is a general molecular regulator in the pathogenesis of ICH, and it is not uniformly beneficial or detrimental; rather, its protective or damaging effects depend greatly on the context, time and location of expression. In the acute stage, pathologically high VEGF promotes BBB disruption and vasogenic oedema by strengthening VEGFR-2/Src signaling under thrombin’s influence to cause coagulopathy. As ICH enters the subacute and chronic stages, VEGF transitions to a reparative function, including VEGFR-2/PI3K/Akt–eNOS-mediated angiogenesis, VEGF-C/VEGFR-3-driven glymphatic clearance, and JAK2/STAT3-coupled neurovascular repair.

The VEGF signaling network has some conceptual advantages over the previous neuroprotective strategies for ICH. Earlier approaches, such as glutamate receptor antagonists, calcium channel blockers and free radical scavengers, primarily focused on a single downstream mediator of injury and failed to address the dynamic, phase-dependent pathophysiology of ICH. VEGF is a constitutive synchroniser that can connect the phase of acute injury with the phase of chronic recovery through dual-receptor mechanisms. The first translational problem is the BBB: Although ICH transiently increases BBB permeability, this disruption is heterogeneous, time-limited, and mostly pathological rather than suitable for controlled drug delivery. To address this problem, engineering delivery platforms such as AAV vectors with inducible promoters, BBB-penetrating nanoliposomes, and stimulus-responsive fusion proteins can be used to achieve spatiotemporally controlled activation of the pathway only in the perihematomal area during the recovery phase.

Based on the above mechanisms and translation effects, we propose that a dual-function approach is needed for the treatment: reduce acute disruption of the BBB by targeting VEGFR-2/Src simultaneously, and then activate protective angiogenesis via the PI3K/Akt/VEGFR-3 and VEGFR-2 pathways sequentially. To this end, we need to introduce the new developments in receptor-selective pharmacology, precision drug delivery and biomarker-guided patient stratification. In short, VEGF is a general pro-angiogenic factor that regulates all parts of neurovascular repair, and thus, targeted therapies should be developed that are both temporally and dose-sensible. Advancing the paradigm of mechanism-based multi-target neurovascular drugs will likely reduce the huge worldwide burden of ICH.
